# Towards Simplification of Ketogenic Diet in Epilepsy: Effect of Caprylic (C8) and Capric (C10) Acid on the Mitochondrial Respiratory Chain in Murine Hippocampal Neurons In Vitro

**DOI:** 10.3390/nu18020216

**Published:** 2026-01-09

**Authors:** Miriam Rebekka Rühling, Hans Hartmann, Anibh Martin Das

**Affiliations:** Clinic for Paediatric Kidney-, Liver- and Metabolic Diseases and Neuropaediatrics, Hannover Medical School, Carl-Neuberg Str. 1, D-30625 Hannover, Germany; miriam.rebekka.ruehling@tiho-hannover.de (M.R.R.); hartmann.hans@mh-hannover.de (H.H.)

**Keywords:** ketogenic diet, mitochondrial respiratory chain enzymes, medium-chain fatty acids, ß-hydroxybutyrate, pharmacoresistant epilepsy

## Abstract

Background: Pharmacotherapy is the therapeutic mainstay in epilepsy, but in about 30% of patients, the epilepsy is pharmacoresistant. A ketogenic diet (KD) is an alternative therapeutic option. The mechanisms underlying the anti-seizure effect of KD are not fully understood. An enhanced energy metabolism may have a protective effect; C8 and C10 fatty acids were previously shown to activate mitochondrial function in vitro. In the present study, we investigated whether ß-hydroxybutyrate (HOB), C8, C10 or a combination of C8 and C10 fatty acids, which all increase under KD, could activate mitochondrial respiratory chain enzymes in murine hippocampal neurons (HT22). Methods: Cells were incubated for one week in the presence of the different metabolites. Respiratory chain enzyme activities as well as citrate synthase as a mitochondrial marker enzyme were determined spectrophotometrically in these cells. We observed that enzyme activities of complexes I and III, II and III, and IV (cytochrome c-oxidase) and V (ATP synthase) significantly increased in response to incubation with C8 and C10 fatty acids and a combination of both. Results: This activation of the respiratory chain enzymes was not inferior to an incubation with HOB, the key metabolite in KD. The activity of the mitochondrial marker enzyme citrate synthase increased under incubation with the fatty acids, showing that the mitochondrial content increased. Conclusions: In murine hippocampal cells, C8, C10 and combined C8 and C10 fatty acids led to variable increases in activities of mitochondrial respiratory chain enzymes and citrate synthase. This indicates that both C8 and C10 fatty acids may be important for the antiepileptic effect of KD, as they enhance energy production.

## 1. Introduction

Epilepsy affects app. 50 million people worldwide [1]. A recent population-based study in Norway found a cumulative incidence of 0.66% at age 10 years [2].

In most patients with epilepsy, pharmacotherapy is the first-line treatment, but effective pharmacological seizure control can only be achieved in less than 70% of patients [3]. Ketogenic diet (KD) is an established therapeutic approach for patients with pharmacoresistant epilepsy [4,5,6].

Pharmacotherapy modulates the function of neuronal ion channels and neurotransmitters. Several other mechanisms underlying the antiepileptic effect of KD have been suggested. KD is supposed to reduce neuronal excitability [7] and modulate excitatory–inhibitory regulation of key neurotransmitters such as glutamate and GABA [8]. Other hypotheses of the positive effect are modifications of the Bad protein, Wnt/β-catenin signalling pathway, mTOR, and Nrf2 [9,10,11].

The brain has a high basic energy demand which is comparable to the energy demand of leg muscles during marathon running [12]. The bulk of energy is used for neuronal synaptic processes, while homeostasis of ion gradients requires less energy, most of which is derived from mitochondrial oxidative phosphorylation [13]. Epileptic seizures were found to compromise mitochondrial function [13]. Mitochondrial dysfunction may not only result in energy deficiency but dysbalance of neurotransmitters, compromised calcium and redox signalling as well as increased production of reactive oxygen species, which can contribute to neurodegeneration [13] and chronic inflammation [14]. In rats, mitochondrial dysfunction was associated with neuronal death after status epilepticus [15].

Many genetic disorders of energy metabolism, especially mitochondriopathies, glucose transporter defects and long-chain fatty acid oxidation defects, may manifest with epilepsy, underlining the importance of energy metabolism for the integrity of brain function [16].

Epileptic seizures lead to an increased energy demand of neurons with subsequent neuronal damage and an unstable membrane potential [17]. Redressing the neuronal energetic balance by enhanced energy production may be neuroprotective in terms of reducing epileptic seizures [18] and neuronal damage. Energy production can be enhanced by KD as previously suggested [19,20,21,22,23]. Rats which were fed a KD showed an improved mitochondrial function [24,25].

When following a KD, high amounts of fat, small amounts of carbohydrates and adequate amounts of proteins are consumed [26]. Several types of KD have been described [27], such as the classical KD with a fixed lipid-to-carbohydrate ratio, the Atkins diet characterised by a defined (low) amount of carbohydrate intake, and the MCT (medium-chain triglyceride) ketogenic diet [28]. The MCT-KD allows higher carbohydrate intake without impacting efficacy of the diet [29] as MCTs have an increased ketogenic potential [30]. Another type of KD, the Low-Glycaemic-Index treatment (LGIT), allows an even higher carbohydrate intake limited to those carbohydrates with a low glycaemic index. This results in lower ketone concentrations compared to the classical KD and Atkins diet [8].

The high fat content of KD leads to an increased hepatic ketone body production [31] and ketonaemia [26]. This shift has been shown to improve mitochondrial function in extrahepatic tissues, including the brain [32].

It is generally believed that ketone bodies, as the lead compounds of KD [33], mediate the antiepileptic effect. However, KD leads to an increase in other metabolites as well [34]. These include medium-chain fatty acids, namely C8 (caprylic acid) and C10 (capric acid) fatty acids [35]. Indeed, C10 fatty acids have been shown to increase the mitochondrial content in neurons [19,20,21,22,23,24] in vitro, potentially resulting in neuroprotection. Furthermore, it was shown that an MCT diet was able to reduce seizure frequency in an animal model of Dravet syndrome [36]. In human seizure patients, MCT supplementation to KD had a beneficial effect as well [37].

Adherence to KD is challenging as it is unpalatable and may cause gastrointestinal symptoms [38]. ß-hydroxybutyrate (HOB) has similar disadvantages with even stronger gastrointestinal side-effects [38]. Use of C8 and/or C10 fatty acids would improve adherence as these compounds are more palatable than KD and HOB.

We have recently shown that C8 and C10 fatty acids and HOB activated NAD+-dependent sirtuins in cultured hippocampal neurons [21,23]. These enzymes have various physiological functions, such as stimulation of mitochondrial biogenesis, detoxification of reactive oxygen species, and regulation of fatty acid oxidation and respiratory chain enzymes [23]. In a previous pilot study, we already showed that C10 fatty acids activated mitochondrial respiratory chain enzyme activities, while we did not investigate the effects of C8 [21].

We hypothesised that not only HOB but also C8 and C10 fatty acids have a positive effect on the mitochondrial respiratory chain. This prompted us to study the effect of both C8 and C10 fatty acids on mitochondrial respiratory chain enzymes in neurons in comparison to HOB as the lead substance in KD, in cultured HT22 murine hippocampal neuronal cells as a model.

## 2. Materials and Methods

### 2.1. Cell Culture

For this study, HT22 murine hippocampal neuronal cells from an immortalised murine cell line were used (kindly provided by Prof. K. Haastert-Tallini, Institute of Neuroanatomy, Hannover Medical School, Hannover, Germany).

Cells were cultured in T25 culture flasks (Sarstedt AG, Nümbrecht, Germany) using Dulbecco’s Modified Eagle Medium (DMEM) (Thermo Scientific, Waltham, MA, USA), supplemented with 10% (*v*/*v*) foetal bovine serum (Biowest SAS, Nuaillé, France) and 1% (*v*/*v*) penicillin/streptomycin (Sigma-Aldrich, Taufkirchen, Germany) at 37 °C and 5% CO_2_.

In an attempt to mimic a ketogenic/LGI environment (high fatty acids, low glucose) we added 1 g glucose (Thermo Scientific, Waltham, MA, USA) per litre to the medium; the standard concentration of glucose in the culture medium was 4.5 g per litre for HT22 cells. Lower glucose concentrations than 1 g per litre in the medium resulted in reduced growth of HT22 cells [39].

**Figure 1:**
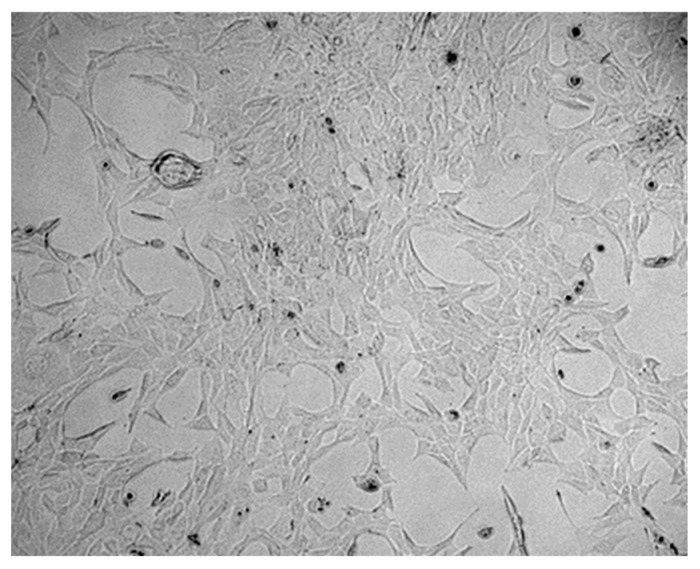
Hippocampal cell culture.

The cells were incubated for 1 week in the presence of metabolites which increase under a KD; media with respective metabolites were changed every other day. At the beginning of experimentation when supplements were added to the medium, the confluence of cells was about 50% (see Figure 1). After one week of incubation, the confluence was approximately 80%. Cells were either incubated without supplements (controls, C), sham-incubated with ethanol (EC, concentration in the medium was 0.5%) which served as the carrier of medium-chain fatty acids, or incubated with C8 (Sigma-Aldrich, Taufkirchen, Germany) (250 µM) or C10 fatty acid (Sigma-Aldrich, Taufkirchen, Germany) (250 µM), or a 1:1 mixture of both (a final concentration of 125 µM, respectively) or HOB (Sigma-Aldrich, Taufkirchen, Germany) (5 mM). These concentrations of HOB and C8 and C10 fatty acids reflect concentrations found during a KD in cerebrospinal fluid (CSF) of patients [40,41].

Cell cultures were incubated in three biological replicates for each incubation condition.

### 2.2. Preparation of Petri Dishes for Measurement

The cell culture medium was removed from the Petri dishes and cells were washed twice with a HEPES buffer. This HEPES buffer contained NaCl (110 mM), KCl (2.6 mM), KH_2_PO_4_ (1.2 mM), MgSO_4_ × 7 H_2_O (1.2 mM), and CaCl_2_ (1 mM, HEPES (Carl Roth GmbH, Karlsruhe, Germany)) (25 mM). Subsequently, cells were incubated in HEPES buffer supplemented with glucose (at a final concentration of 7.5 mM) at room temperature for 15 min.

Cells were then disrupted by sonication twice for 10 s using the Bandelin Sonopuls HD70 sonicator to expose intramitochondrial respiratory chain enzymes. For the subsequent measurement of the protein concentration, 125 µL of cell lysate was pipetted into an Eppendorf tube.

Enzyme assays were performed in triplicate (technical repeats).

### 2.3. Analytical Procedures

For spectrophotometric measurements of enzymatic capacities of complexes I–V of the MRC and CS (a marker of mitochondrial content and quality), a UV spectrophotometer (UV ChemStation 32 software) (Agilent technologies, Santa Clara, CA, USA) was used.

#### 2.3.1. Measurement of Complexes I + III (NADH-Oxidase)

A slight modification of a previously described method was used [42]; Rotenone served as a specific inhibitor. The spectrophotometric measurement was performed at 340 nm wavelength. Measurements were carried out in a cuvette with 850 µL modified buffer (without NADH) (KCl end concentration of 50 mM, Saccharose end concentration of 60 mM, Triethanolamine-HCl end concentration of 50 mM, MgCl_2_ end concentration of 2 mM, EDTA end concentration of 1 mM). Samples were measured by pipetting 850 µL measurement buffer and 5 µL Cytochrome C solution into a cuvette. The measurement was started by adding 150 µL of cell suspension and stopped by adding 10 µL Rotenone.

#### 2.3.2. Measurement of Complexes II + III (Antimycin-Sensitive Succinate–Cytochrome C Reductase)

Spectrophotometric measurements were performed at a wavelength of 550 nm and 37 °C according to Stumpf and Parks [43] with antimycin as a specific inhibitor. Measurements were carried out in a buffer containing 750 µL KH_2_PO_4_ solution (50 mM) + 10 µL NaN_3_ (250 mM). Cytochrome C (cyt c) and Rotenone were added and the reaction was started by adding 200 µL cell suspension and succinate; finally the reaction was stopped by antimycin.

#### 2.3.3. Measurement of Complex IV (Cytochrome C Oxidase)

Spectrophotometric measurements were performed at a wavelength of 550 nm and 37 °C using a slight modification of a method previously described [44]. Two cuvettes were filled with the reaction buffer containing 10 mM KH_2_PO_4_ (pH 7.0) and ferrocytochrome C (Sigma). Differential spectroscopy was performed. In the reference cuvette, ferrocytochrome C was oxidised by potassium ferricyanide. The reaction was initiated by adding cell lysate to the measuring cuvette. Finally, the reaction was stopped by adding potassium ferricyanide to the measuring cuvette.

#### 2.3.4. Measurement of Complex V (ATPase Assay)

Spectrophotometric measurements were performed at a wavelength of 340 nm and a temperature of 37 °C using a slight modification of a method previously described [45,46] with oligomycin as a specific inhibitor. The assay buffer contained 60 mM sucrose, 500 mM triethanolamine-HCl, 50 mM potassium chloride, 4 mM magnesium chloride, 2 mM ATP, 2 mM EGTA and potassium cyanide, pH 8.0, and 100 µM NADH. Pyruvate kinase and lactate dehydrogenase served as coupling enzymes. The reaction was started by adding the cell lysate and was finally stopped by oligomycin.

#### 2.3.5. Measurement of Citrate Synthase

Citrate synthase is a marker of the mitochondrial amount and quality. The spectrophotometric measurement was performed at a wavelength of 232 nm [21]. Tris buffer was pipetted into a quartz cuvette; oxalate and acetyl-CoA were added and the reaction was started by adding 100 µL of cell suspension.

#### 2.3.6. Protein Measurement

Trichloroacetic acid (TCA) precipitation was performed to separate proteins from the cell lysate for quantification [47]. Subsequently, the bicinchoninic acid (BCA) assay kit (Thermo Fischer Scientific Inc., USA) was used to quantify the protein content [21].

### 2.4. Materials/Reagents

All reagents and chemicals were purchased from Sigma Aldrich, Taufkirchen, Germany: cytochrome C, rotenone, succinate, antimycin, cytochrome C, potassium ferricyanide, oligomycin, oxalate and acetyl-CoA.

### 2.5. Statistical Analysis

The results were statistically evaluated and analysed using SAS^®^ Enterprise Guide^®^ (Version 7.1) computer software (SAS Institute Inc., Cary, NC, USA). Analysis of variance (ANOVA) was used to compare the six incubation groups to controls. Results are shown as mean + SD; *p* < 0.05 was regarded as significant and *p* < 0.01 as highly significant.

## 3. Results

### 3.1. Complexes I + III (NADH-Oxidase)

**Figure 2:**
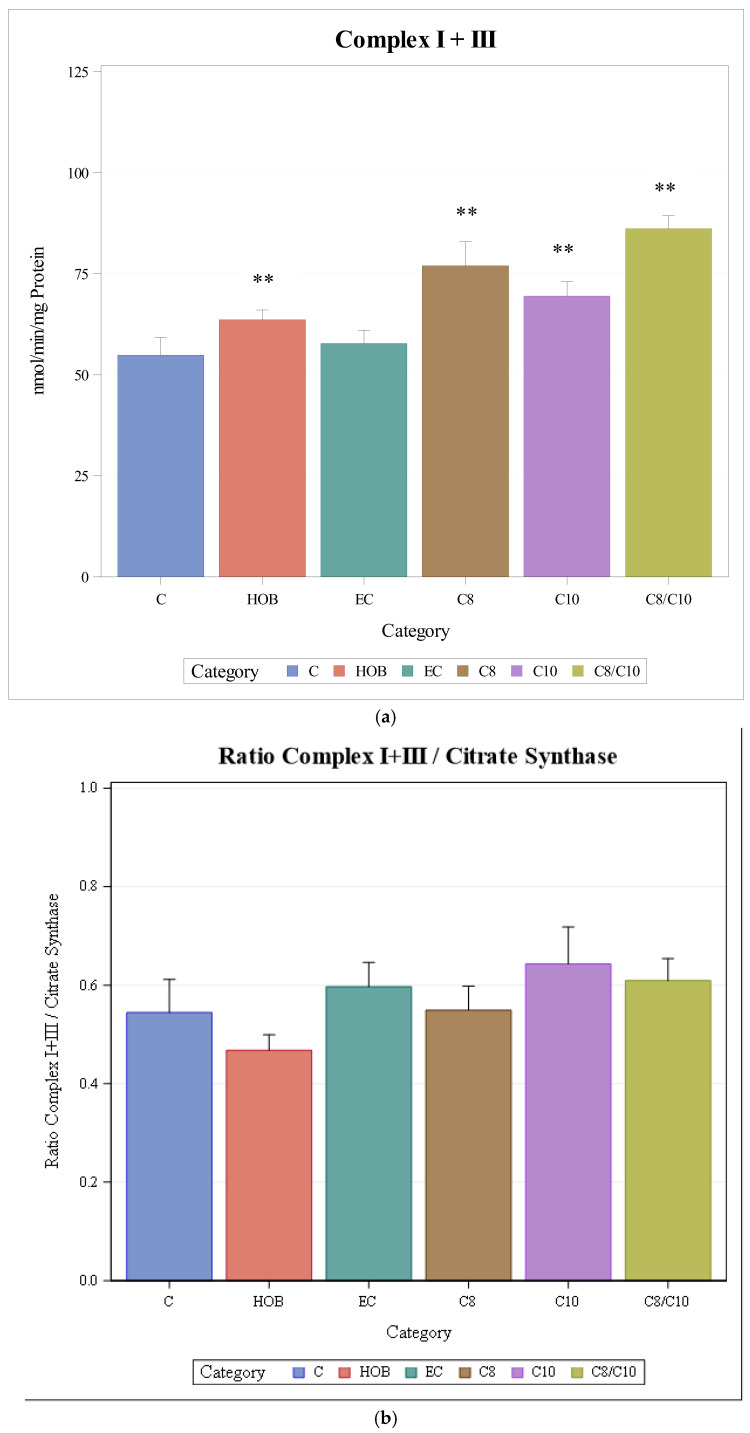
(**a**): Respiratory chain enzyme activity of complexes I + III under different incubation conditions (mean + SD), *n* = 3, * *p* < 0.05, ** *p* < 0.01 (vs. C). C = control, HOB = ß-hydroxybutyrate, EC = ethanol control, C8 = C8 fatty acid, C10 = C10 fatty acid, C8/C10 = C8 + C10 fatty acid. (**b**): Respiratory chain enzyme activity of complexes I + III normalised to CS under different incubation conditions (mean + SD), *n* = 3. C = control, HOB = ß-hydroxybutyrate, EC = ethanol control, C8 = C8 fatty acid, C10 = C10 fatty acid, C8/C10 = C8 + C10 fatty acid.

The activity of complexes I + III increased significantly when cells were incubated with C8, C10, and C8/10 fatty acids or HOB. Ethanol as the solvent for fatty acids did not have an effect on the enzyme activity (Figure 2a).

When complex I + III activities were normalised to citrate synthase activity (as a mitochondrial marker enzyme), no significant differences between different incubation conditions could be observed (Figure 2b).

### 3.2. Complexes II + III (Antimycin-Sensitive Succinate–Cytochrome C Reductase)

**Figure 3:**
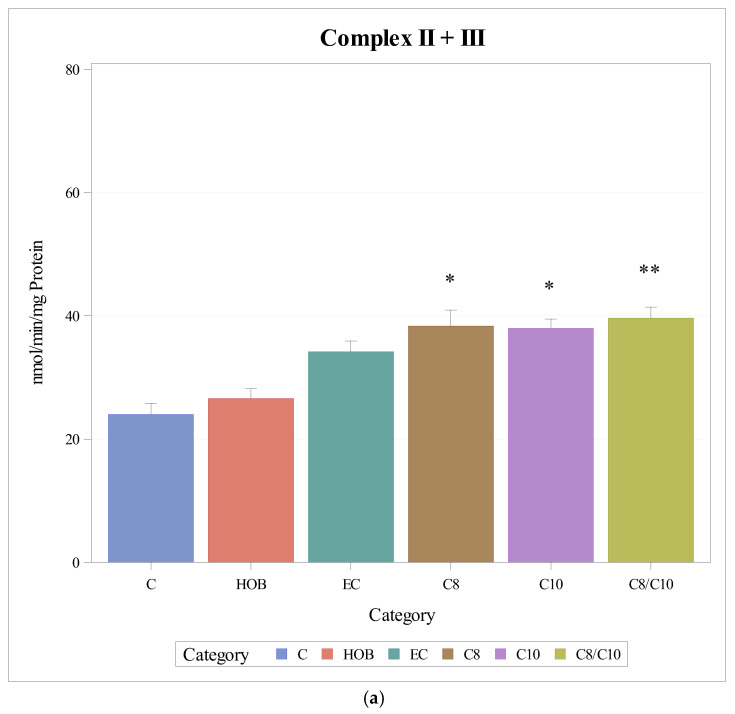

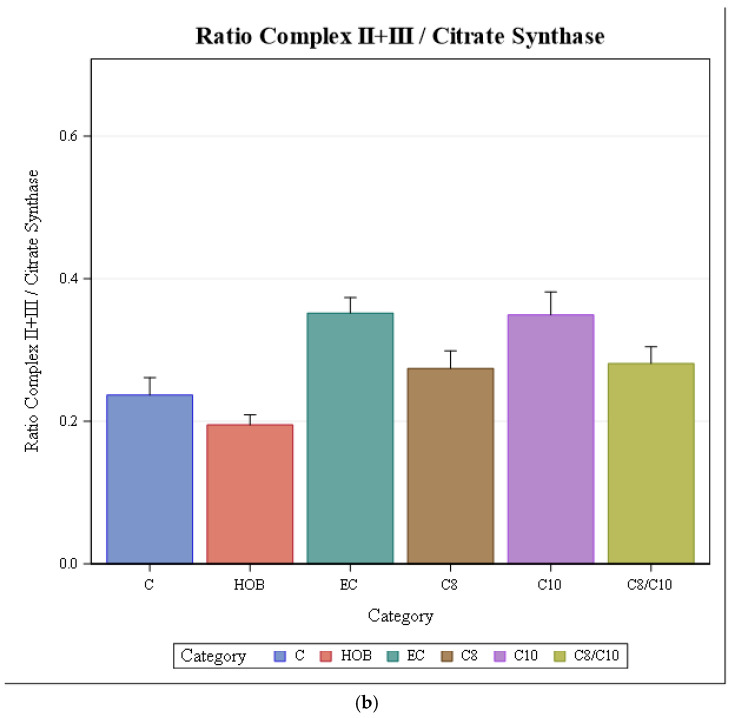
(**a**): Respiratory chain enzyme activity of complexes II + III under different incubation conditions (mean + SD), *n* = 3, * *p* < 0.05 ** *p* < 0.01 (vs. C). C = control, HOB = ß-hydroxybutyrate, EC = ethanol control, C8 = C8 fatty acid, C10 = C10 fatty acid, C8/C10 = C8 + C10 fatty acid. (**b**): Respiratory chain enzyme activity of complexes II + III normalised to CS under different incubation conditions (mean + SD), *n* = 3. C = control, HOB = ß-hydroxybutyrate, EC = ethanol control, C8 = C8 fatty acid, C10 = C10 fatty acid, C8/C10 = C8 + C10 fatty acid.

The respiratory chain enzyme activities of complexes II + III increased significantly when cells were incubated with C8, C10 and C8/10 fatty acids. Ethanol did not impact enzyme activity. HOB did not have a significant effect on enzyme activity (Figure 3a).

When complex II + III activities under the different incubation conditions were normalised to citrate synthase, no differences could be observed (Figure 3b).

### 3.3. Complex IV (Cytochrome C Oxidase)

**Figure 4:**
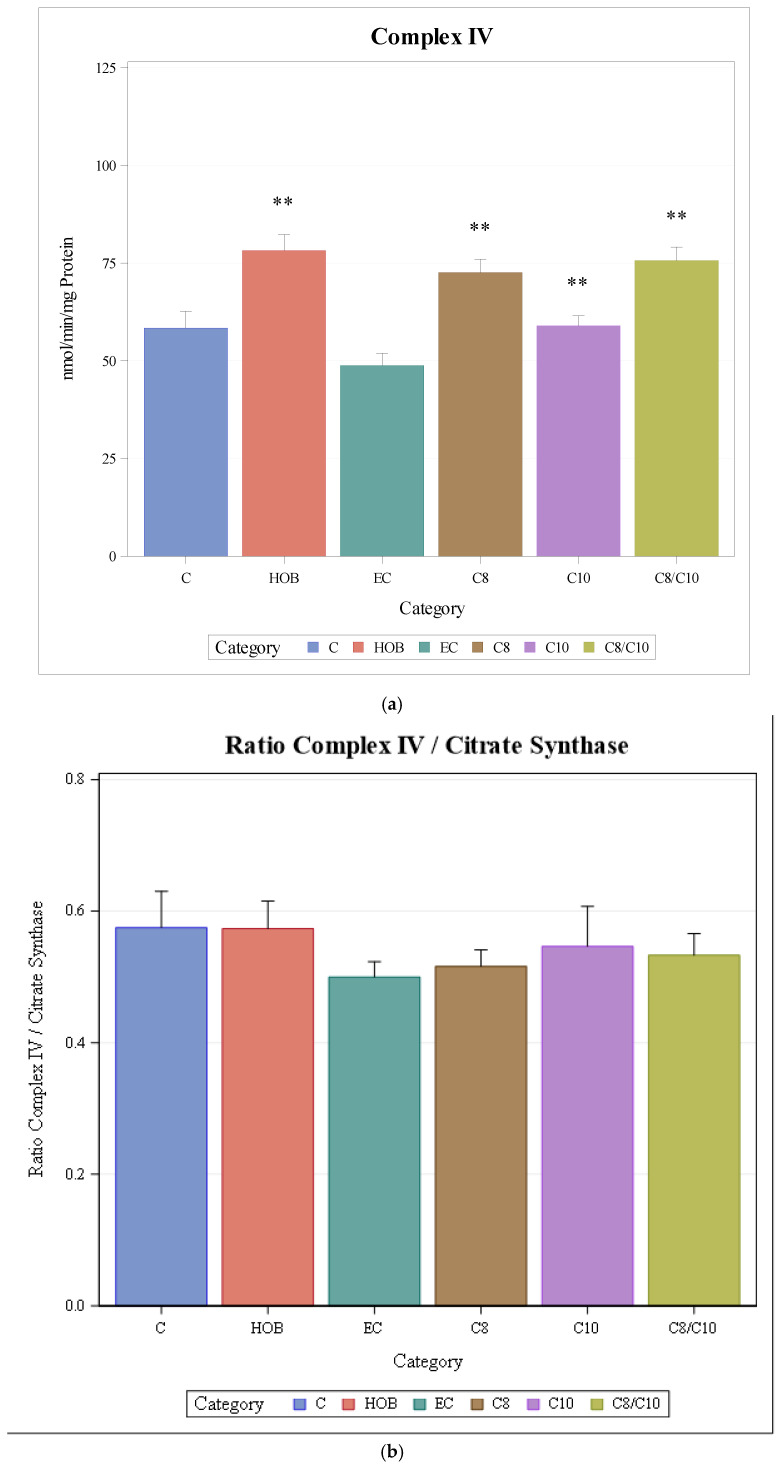
(**a**): Respiratory chain enzyme activity of complex IV under different incubation conditions seen above (mean + SD), *n* = 3, * *p* < 0.05, ** *p* < 0.01 (vs. C). C = control, HOB = ß-hydroxybutyrate, EC = ethanol control, C8 = C8 fatty acid, C10 = C10 fatty acid, C8/C10 = C8 + C10 fatty acid. (**b**): Respiratory chain enzyme activity of complex IV under different incubation conditions normalised to citrate synthase (mean + SD), *n* = 3. C = control, HOB = ß-hydroxybutyrate, EC = ethanol control, C8 = C8 fatty acid, C10 = C10 fatty acid, C8/C10 = C8 + C10 fatty acid.

The respiratory chain enzyme activity of complex IV increased significantly when cells were incubated with C8, C10, and C8/10 fatty acids and HOB compared to controls and ethanol controls, respectively (Figure 4a).

When complex IV activities under the different incubation conditions were normalised to citrate synthase as the mitochondrial marker enzyme, no changes under the different incubation conditions could be observed (Figure 4b).

### 3.4. Complex V (ATP Synthase)

**Figure 5:**
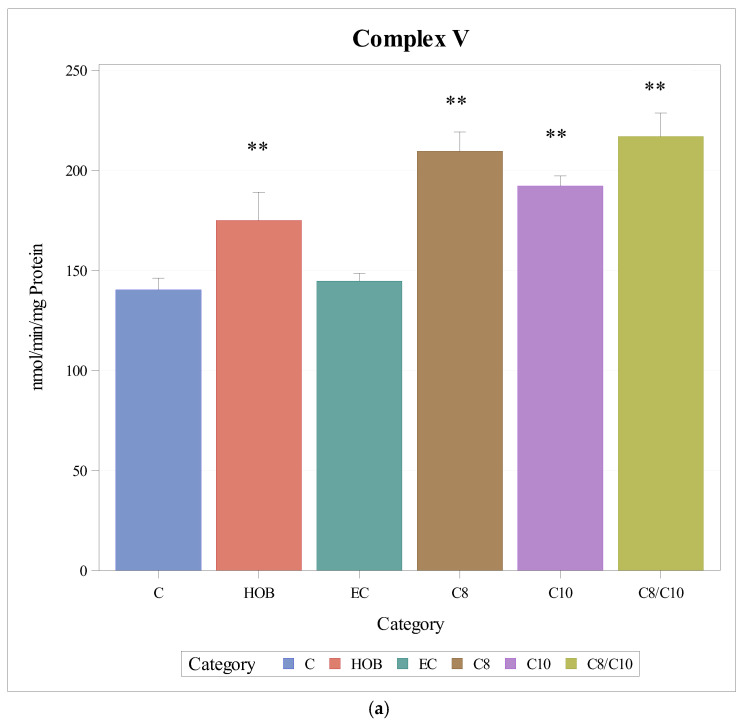

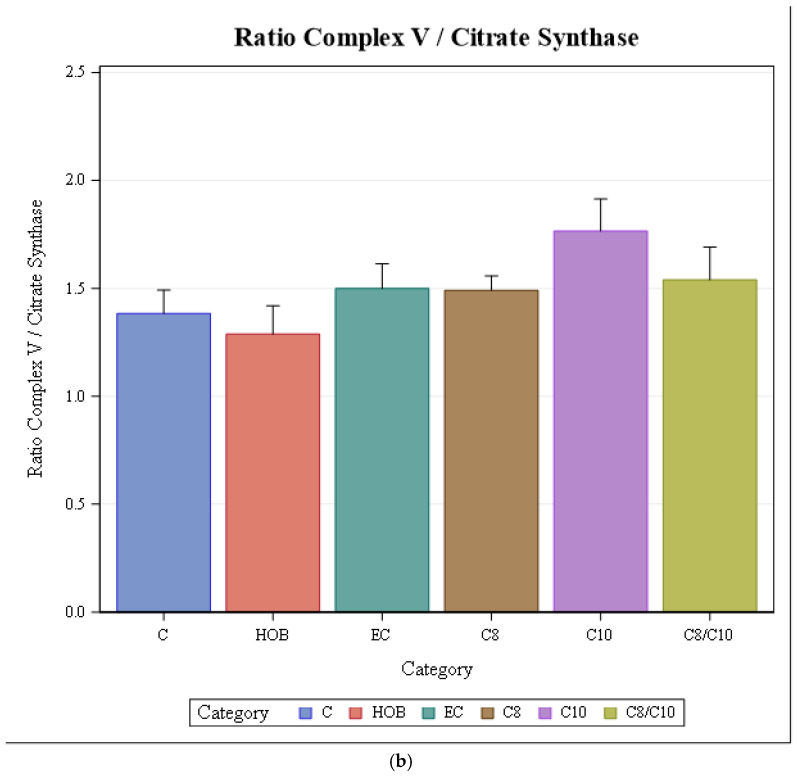
(**a**): Respiratory chain enzyme activity of complex V under different incubation conditions (mean + SD), *n* = 3, * *p* < 0.05, ** *p* < 0.01 (vs. C). C = control, HOB = ß-hydroxybutyrate, EC = ethanol control, C8 = C8 fatty acid, C10 = C10 fatty acid, C8/C10 = C8 + C10 fatty acid. (**b**): Respiratory chain enzyme activity of complex V normalised to citrate synthase under different incubation conditions (mean + SD), *n* = 3, * *p* < 0.05 (vs. C). C = control, HOB = ß-hydroxybutyrate, EC = ethanol control, C8 = C8 fatty acid, C10 = C10 fatty acid, C8/C10 = C8 + C10 fatty acid.

The respiratory chain enzyme activities of complex V increased significantly when cells were incubated with C8, C10, and C8/10 fatty acids and HOB compared to controls and ethanol controls (Figure 5a).

When complex V activities were normalised to citrate synthase, a significant increase was observed only under the incubation with C10 (Figure 5b).

### 3.5. Citrate Synthase

**Figure 6:**
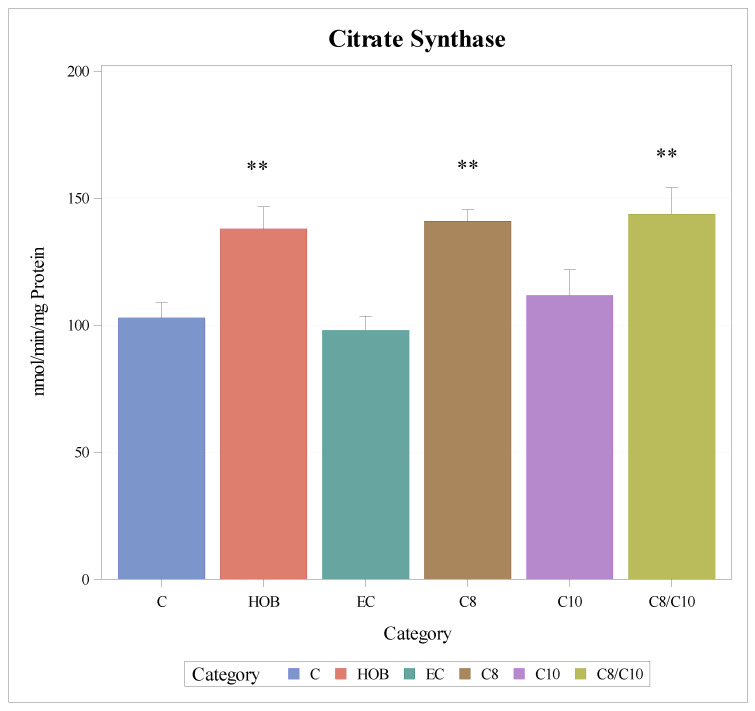
Enzyme activity of citrate synthase under different incubation conditions (mean + SD), *n* = 3 ** *p* < 0.01 (vs. C). C = control, HOB = ß-hydroxybutyrate, EC = ethanol control, C8 = C8 fatty acid, C10 = C10 fatty acid, C8/C10 = C8 + C10 fatty acid.

The enzyme activity of citrate synthase as a mitochondrial marker enzyme increased significantly when cells were incubated with C8 and C8/10 fatty acids and HOB (Figure 6).

## 4. Discussion

The brain has a high basic energy demand which is comparable to the energy demand of leg muscles during marathon running [18]. The bulk of energy is used for neuronal synaptic processes, while homeostasis of ion gradients requires less energy, most of which is derived from mitochondrial oxidative phosphorylation [13].

Many genetic disorders of energy metabolism, especially mitochondriopathies and long-chain fatty acid oxidation defects, may manifest with epilepsy, showing the importance of energy metabolism for the integrity of the brain [16].

Epileptic seizures lead to an increased energy demand of neurons with subsequent neuronal damage and an unstable membrane potential [17]. Mitochondrial dysfunction in epilepsy can contribute to energy depletion in the brain [13]. The energy balance can be redressed by increased energy production mediated by KD, as previously suggested [19,20,21,22,23]. KD leads to an increase in ATP, which supports hyperpolarisation of the neuronal membrane [18]. Apart from compromised energy production, mitochondrial dysfunction may result in dysbalance of neurotransmitters, compromised calcium handling and redox signalling as well as increased production of reactive oxygen species, which can contribute to neurodegeneration [13] and chronic inflammation [14].

Previous studies showed that the KD has a strong influence on energy metabolism. Here, the pattern of gene expression in the hippocampus of rats fed a KD was analysed. They concluded that an increased mitochondrial biogenesis together with regulation of various genes involved in energy metabolism mediated the anticonvulsant effect in rats which were fed a KD [24,25]. The role of mitochondria might be particularly relevant in status epilepticus. In rats, it was shown that mitochondrial dysfunction was associated with neuronal death after status epilepticus [15].

Thus, the neuronal energy balance is important in epilepsy; enhanced energy production may be neuroprotective. The antiepileptic effect of KD may be partly mediated by enhancing mitochondrial energy production. C8 and C10 fatty acid concentrations were previously reported to increase significantly in blood of children with pharmaco-resistant epilepsy undergoing a KD [34]. Therefore, they are promising candidates which can enhance energy production.

This prompted us to assess the effect of these medium-chain fatty acids on energy metabolism of murine hippocampal neurons in vitro. The concentrations of HOB and C8 and C10 fatty acids used in our study reflect concentrations found during a KD in cerebrospinal fluid (CSF) of patients [40,41]. In a previous study, we observed an activation of sirtuin enzyme activities in cultured HT22 cells after incubation with C8 and/or C10 fatty acids [23]. Sirtuins have various subcellular localisations such as mitochondria, nucleus and cytoplasm. They regulate various physiological functions such as mitochondrial biogenesis, detoxification of reactive oxygen species, fatty acid oxidation, and regulation of respiratory chain enzymes, ATP synthase and citrate synthase [48].

We hypothesised that C8 and/or C10 fatty acids have an activating effect on respiratory chain enzymes and energy metabolism in hippocampal HT22 cells. This may redress the balance between enhanced energy consumption and energy production during epileptic seizures. Indeed, we observed in the present study that enzyme activities of complexes I + III (NADH-oxidase), II + III (antimycin-sensitive succinate–cytochrome C reductase) and IV (cytochrome c-oxidase) significantly increased in response to incubation with C8 and C10 fatty acids and a combination of both fatty acids. The activity of complex V (ATP synthase) only increased after incubation with C8 and a combination of C8 and C10 fatty acids. This activation of the respiratory chain enzymes was not inferior to an incubation with HOB, the ‘gold standard’ and key metabolite in KD. The activity of the mitochondrial marker enzyme citrate synthase increased under incubation with C8 and a combination of C8 and C10 fatty acids as well as HOB, suggesting that the mitochondrial content increased. Sirtuins could play an important role in mitochondrial biogenesis induced by medium-chain fatty acids by the activation of PGC-1 alpha [13].

There seems to be no posttranslational modification under incubation of HT22 cells with C8 and/or C10 fatty acids, as respiratory chain enzyme activities remained unchanged when normalised to citrate synthase. An exception was complex V (ATP synthase) activity. When cells were incubated with C10 fatty acid, the enzyme activity of complex V increased after normalisation to citrate synthase. This could be the result of posttranslational modification. On the other hand, we have previously shown that the ATP synthase in rat cardiomyocytes and other tissues is actively regulated in response to energy demand [49]. The calcium-binding inhibitor protein (CaBI) and the inhibitor protein IF1 have been shown to be regulatory elements of ATP synthase. We reported previously that ATP synthase is dysregulated in fibroblasts from children with the neuronal disorder neuronal ceroid lipofuscinosis [50] and postulated that this altered regulation can be found in the CNS as well. If active regulation of ATP synthase can be found in HT22 cells, upregulation of the enzyme may be mediated by abnormal mitochondrial handling of calcium under C10 fatty acid incubation.

In a previous study, it was shown that C8 fatty acids had a weaker antiepileptic effect compared to C10 fatty acids, which was different from our findings. This was explained by a compromised carnitine-dependent mitochondrial uptake of C10 fatty acids, which resulted in the extramitochondrial accumulation of C10 compounds [51]. In terms of sirtuin activation and mitochondrial function we did not observe differences between C8 and C10 fatty acids. This contrasts with a previous study by Hughes in SH-SY5Y cells that found that only C10, not C8, improved citrate synthase activity [19]. These discrepancies might be explained by the different cell models that were used. It should be noted that the HT22 cells used in our study are sensitive to glutamate excitotoxicity, and hence a valid model for epilepsy, while the neuroblastoma cell lines used in other experiments serve as a valid model for neurodegenerative diseases. Carnitine-dependent mitochondrial uptake of C10 fatty acids may be different between the two cell types.

If the results of our study can be translated to clinical medicine in patients with epilepsy this may explain the beneficial effect of KD in epilepsy and may have implications on alternative diets. As medium-chain fatty acids were not inferior to HOB in terms of mitochondrial biogenesis and thus upregulation of respiratory chain enzymes, a simplified alternative to KD could be the supplementation of C8 and C10 fatty acids. Astrocytes are able to metabolise medium-chain fatty acids which cross the blood–brain barrier [52]. Therefore, the effects of MCT supplementation on this cell type also warrant further investigation.

Our study has some limitations. Experiments were only performed in cultured neurons in vitro. It is not clear whether the changes observed in energy metabolism can be found in animal models or humans in vivo. In a previous study we observed changes in sirtuins in blood from patients with pharmaco-resistant epilepsy undergoing a modified Atkins diet [23]. We plan to study the effect of C8 and C10 fatty acid supplementation on sirtuins in blood from patients with epilepsy. Furthermore, we only investigated the impact of medium-chain fatty acids on HT22 neuronal cells. It is not clear whether C8 and C10 fatty acids can modulate energy metabolism in other types of cells as well. In a previous study, we were able to detect alterations of sirtuins in blood from patients with epilepsy undergoing a modified Atkins diet [23]. Furthermore, we measured sirtuins in blood from exercising individuals under different diets and found differences in the response to exercise [53]. These results suggest that the modulation of energy metabolisms by medium-chain fatty acids is not limited to neurons in vivo. The amount of respiratory chain complex proteins will be studied in future studies using Western Blotting.

Beyond pharmacoresistant epilepsy, supplementation of C8 and C10 fatty acids as a booster of mitochondrial function may be useful in inborn errors of mitochondrial energy metabolism like mitochondriopathies and fatty acid oxidation defects; urea cycle defects like ornithine transcarbamylase (OTC) and carbamylphosphate synthase (CPS) deficiency, etc., may benefit as well.

Unlike long-chain fatty acids, C8 and C10 fatty acids are absorbed quickly from the intestine and transported via the portal vein directly to the liver rather than through chylomicron-mediated lymphatic transport. This leads to rapid increases in plasma levels of these fatty acids after ingestion of MCTs [54]. Concentrations of these fatty acids are not substantially affected by meals, indicating sustained plasma presence during metabolic processing [30].

Two commercial products containing C8 and C10 fatty acids, namely KetoEpi^®^ and K.vita^®^, are available as alternatives to KD for the treatment of epileptic patients not responding to pharmacotherapy. On the other hand, other MCT products may serve as a source of C8 and C10 fatty acids, but the ratio of both fatty acids varies in different products.

## 5. Conclusions

Epilepsy is characterised by mitochondrial dysfunction and energy deficit in the brain. The results of this study show that various metabolites, namely C8 and C10 fatty acids and HOB, which all increase during KD, have a stimulating effect on respiratory chain enzymes and mitochondrial biogenesis in cultured hippocampal neurons from mice. Thus, they may be able to redress the energetic balance in epilepsy. The improved energetic balance may be a mechanism underlying the beneficial effect of KD in epilepsy. The medium-chain fatty acids were not inferior to HOB, the lead compound in KD. C8 and C10 fatty acids are therefore promising metabolites, which may contribute to the therapeutic effects of the KD and serve as an alternative option in the treatment of epilepsy.

## Data Availability

The original contributions presented in this study are included in the article. Further inquiries can be directed to the corresponding author.

## References

[1] World Health Organization (2019). Epilepsy: A Public Health Imperative.

[2] Aaberg K.M., Gunnes N., Bakken I.J., Søraas C.L., Berntsen A., Magnus P., Lossius M.I., Stoltenberg C., Chin R., Surén P. (2017). Incidence and Prevalence of Childhood Epilepsy: A Nationwide Cohort Study. Pediatrics.

[3] Dhamija R., Eckert S., Wirrell E. (2013). Ketogenic Diet. Can. J. Neurol. Sci..

[4] Borowicz-Reutt K., Krawczyk M., Czernia J., Łuszczki J.J. (2024). Ketogenic Diet in the Treatment of Epilepsy. Nutrients.

[5] Winesett S.P., Bessone S.K., Kossoff E.H. (2015). The Ketogenic Diet in Pharmacoresistant Childhood Epilepsy. Expert Rev. Neurother..

[6] Kossoff E.H., Zupec-Kania B.A., Auvin S., Ballaban-Gil K.R., Bergqvist A.G.C., Blackford R., Buchhalter J.R., Caraballo R.H., Cross J.H., Dahlin M.G. (2018). Optimal Clinical Management of Children Receiving Dietary Therapies for Epilepsy: Updated Recommendations of the International Ketogenic Diet Study Group. Epilepsia Open.

[7] Lutas A., Yellen G. (2013). The Ketogenic Diet: Metabolic Influences on Brain Excitability and Epilepsy. Trends Neurosci..

[8] Verrotti A., Iapadre G., Di Francesco L., Zagaroli L., Farello G. (2020). Diet in the Treatment of Epilepsy: What We Know So Far. Nutrients.

[9] Giménez-Cassina A., Martínez-François J.R., Fisher J.K., Szlyk B., Polak K., Wiwczar J., Tanner G.R., Lutas A., Yellen G., Danial N.N. (2012). BAD-Dependent Regulation of Fuel Metabolism and KATP Channel Activity Confers Resistance to Epileptic Seizures. Neuron.

[10] Rubio C., Rosiles-Abonce A., Trejo-Solís C., Rubio-Osornio M., Mendoza C., Custodio V., Martínez-Lazcano J.C., González E., Paz C. (2017). Increased Signaling of the Wnt/β-Catenin Pathway and Presence of Apoptosis in the Cerebellum of Kindled Rats. CNS Neurol. Disord. Drug Targets.

[11] McDaniel S.S., Rensing N.R., Thio L.L., Yamada K.A., Wong M. (2011). The Ketogenic Diet Inhibits the Mammalian Target of Rapamycin (mTOR) Pathway. Epilepsia.

[12] Attwell D., Laughlin S.B. (2001). An Energy Budget for Signaling in the Grey Matter of the Brain. J. Cereb. Blood Flow Metab..

[13] Zsurka G., Kunz W.S. (2015). Mitochondrial Dysfunction and Seizures: The Neuronal Energy Crisis. Lancet Neurol..

[14] Kim M.E., Lim Y., Lee J.S. (2025). Mitochondrial dysfunction and metabolic reprogramming in chronic inflammatory diseases: Molecular insights and therapeutic opportunities. Curr. Issues Mol. Biol..

[15] Cock H.R., Tong X., Hargreaves I.P., Heales S.J.R., Clark J.B., Patsalos P.N., Thom M., Groves M., Schapira A.H.V., Shorvon S.D. (2002). Mitochondrial Dysfunction Associated with Neuronal Death Following Status Epilepticus in Rat. Epilepsy Res..

[16] Milder J., Patel M. (2012). Modulation of Oxidative Stress and Mitochondrial Function by the Ketogenic Diet. Epilepsy Res..

[17] Bough K.J., Rho J.M. (2007). Anticonvulsant Mechanisms of the Ketogenic Diet. Epilepsia.

[18] Masino S.A., Li T., Theofilas P., Sandau U.S., Ruskin D.N., Fredholm B.B., Geiger J.D., Aronica E., Boison D. (2011). A Ketogenic Diet Suppresses Seizures in Mice through Adenosine A1 Receptors. J. Clin. Investig..

[19] Hughes S.D., Kanabus M., Anderson G., Hargreaves I.P., Rutherford T., O’Donnell M., Cross J.H., Rahman S., Eaton S., Heales S.J.R. (2014). The Ketogenic Diet Component Decanoic Acid Increases Mitochondrial Citrate Synthase and Complex I Activity in Neuronal Cells. J. Neurochem..

[20] Kanabus M., Fassone E., Hughes S.D., Bilooei S.F., Rutherford T., O’Donnell M., Heales S.J.R., Rahman S. (2016). The Pleiotropic Effects of Decanoic Acid Treatment on Mitochondrial Function in Fibroblasts from Patients with Complex I Deficient Leigh Syndrome. J. Inherit. Metab. Dis..

[21] Dabke P., Das A.M. (2020). Mechanism of Action of Ketogenic Diet Treatment: Impact of Decanoic Acid and β-Hydroxybutyrate on Sirtuins and Energy Metabolism in Hippocampal Murine Neurons. Nutrients.

[22] Augustin K., Khabbush A., Williams S., Eaton S., Orford M., Cross J.H., Heales S.J.R., Walker M.C., Williams R.S.B. (2018). Mechanisms of Action for the Medium-Chain Triglyceride Ketogenic Diet in Neurological and Metabolic Disorders. Lancet Neurol..

[23] Rühling M.R., Hartmann H., Das A.M. (2024). Simplification of Dietary Treatment in Pharmacoresistant Epilepsy: Impact of C8 and C10 Fatty Acids on Sirtuins of Neuronal Cells In Vitro. Nutrients.

[24] DeVivo D.C., Leckie M.P., Ferrendelli J.S., McDougal D.B. (1978). Chronic Ketosis and Cerebral Metabolism. Ann. Neurol..

[25] Bough K.J., Wetherington J., Hassel B., Pare J.F., Gawryluk J.W., Greene J.G., Shaw R., Smith Y., Geiger J.D., Dingledine R.J. (2006). Mitochondrial Biogenesis in the Anticonvulsant Mechanism of the Ketogenic Diet. Ann. Neurol..

[26] Rho J.M., Boison D. (2022). The Metabolic Basis of Epilepsy. Nat. Rev. Neurol..

[27] Ferraris C., Guglielmetti M., Neri L.C.L., Allehdan S., Albasara J.M.M., Alawadhi H.H.F., Trentani C., Perna S., Tagliabue A. (2023). A Review of Ketogenic Dietary Therapies for Epilepsy and Neurological Diseases: A Proposal to Implement an Adapted Model to Include Healthy Mediterranean Products. Foods.

[28] Gano L.B., Patel M., Rho J.M. (2014). Ketogenic Diets, Mitochondria, and Neurological Diseases. J. Lipid Res..

[29] Neal E.G., Chaffe H., Schwartz R.H., Lawson M.S., Edwards N., Fitzsimmons G., Whitney A., Cross J.H. (2009). A Randomized Trial of Classical and Medium-Chain Triglyceride Ketogenic Diets in the Treatment of Childhood Epilepsy. Epilepsia.

[30] St-Pierre V., Vandenberghe C., Lowry C.M., Fortier M., Castellano C.A., Wagner R., Cunnane S.C. (2019). Plasma Ketone and Medium-Chain Fatty Acid Response in Humans Consuming Different Medium-Chain Triglycerides during a Metabolic Study Day. Front. Nutr..

[31] Rogawski M.A., Löscher W., Rho J.M. (2016). Mechanisms of Action of Antiseizure Drugs and the Ketogenic Diet. Cold Spring Harb. Perspect. Med..

[32] Sullivan P.G., Rippy N.A., Dorenbos K., Concepcion R.C., Agarwal A.K., Rho J.M. (2004). The Ketogenic Diet Increases Mitochondrial Uncoupling Protein Levels and Activity. Ann. Neurol..

[33] Simeone T.A., Simeone K.A., Stafstrom C.E., Rho J.M. (2018). Do Ketone Bodies Mediate the Anti-Seizure Effects of the Ketogenic Diet?. Neuropharmacology.

[34] Akiyama M., Akiyama T., Saigusa D., Hishinuma E., Matsukawa N., Shibata T., Tsuchiya H., Mori A., Fujii Y., Mogami Y. (2023). Comprehensive Study of Metabolic Changes Induced by a Ketogenic Diet Therapy Using GC/MS- and LC/MS-Based Metabolomics. Seizure.

[35] Haidukewych D., Forsythe W.I., Sills M. (1982). Monitoring Octanoic and Decanoic Acids in Plasma from Children with Intractable Epilepsy Treated with Medium-Chain Triglyceride Diet. Clin. Chem..

[36] Jancovski N., Baldwin T., Orford M., Li M., Jones G.D., Burbano L.E., Rutherford T., Reid C., Heales S., Eaton S. (2021). Protective Effects of Medium Chain Triglyceride Diet in a Mouse Model of Dravet Syndrome. Epilepsia.

[37] Shin H.J., Ryu S., Lee N., Lee E., Ko A., Kang H.C., Lee J.S., Kim S.H., Kim H.D. (2025). Decanoic Acid-Enriched Ketogenic Diet in Refractory Epilepsy. Front. Neurol..

[38] Huttenlocher P.R., Wilbourn A.J., Signore J.M. (1971). Medium-Chain Triglycerides as a Therapy for Intractable Childhood Epilepsy. Neurology.

[39] Dabke P., Brogden G., Naim H.Y., Das A.M. (2020). Ketogenic Diet: Impact on Cellular Lipids in Hippocampal Murine Neurons. Nutrients.

[40] Zhu H., Bi D., Zhang Y., Kong C., Du J., Wu X., Wei Q., Qin H. (2022). Ketogenic Diet for Human Diseases: The Underlying Mechanisms and Potential for Clinical Implementations. Signal Transduct. Target. Ther..

[41] Omar S.H. (2019). Mediterranean and MIND Diets Containing Olive Biophenols Reduce the Prevalence of Alzheimer’s Disease. Int. J. Mol. Sci..

[42] Fischer J.C., Ruitenbeek W., Trijbels J.M.F., Veerkamp J.H., Stadhouders A.M., Sengers R.C.A., Janssen A.J.M. (1986). Estimation of NADH Oxidation in Human Skeletal Muscle Mitochondria. Clin. Chim. Acta.

[43] Haas R., Chir B., Stumpf D.A., Parks J.K., Eguren L. (1981). Inhibitory Effects of Sodium Valproate on Oxidative Phosphorylation. Neurology.

[44] Wharton D., Tzagoloff A. (1967). Cytochrome c Oxidase from Beef Heart Mitochondria. Methods Enzymol..

[45] Das A.M., Harris D.A. (1990). Regulation of the Mitochondrial ATP Synthase in Intact Rat Cardiomyocytes. Biochem. J..

[46] Rosing J., Harris D.A., Kemp A., Slater E.C. (1975). Nucleotide-Binding Properties of Native and Cold-Treated Mitochondrial ATPase. Biochim. Biophys. Acta.

[47] Bensadoun A., Weinstein D. (1976). Assay of Proteins in the Presence of Interfering Materials. Anal. Biochem..

[48] Wasserfurth P., Nebl J., Rühling M.R., Shammas H., Bednarczyk J., Koehler K., Boßlau T.K., Krüger K., Hahn A., Das A.M. (2021). Impact of Dietary Modifications on Plasma Sirtuins 1, 3 and 5 in Older Overweight Individuals Undergoing 12 Weeks of Circuit Training. Nutrients.

[49] Lin T.Y., Liu H.W., Hung T.M. (2021). The Ketogenic Effect of Medium-Chain Triacylglycerides. Front. Nutr..

[50] Das A.M., Kohlschütter A. (1996). Decreased Activity of the Mitochondrial ATP-Synthase in Fibroblasts from Children with Late-Infantile and Juvenile Neuronal Ceroid Lipofuscinosis. J. Inherit. Metab. Dis..

[51] Khabbush A., Orford M., Tsai Y.C., Rutherford T., O’Donnell M., Eaton S., Heales S.J.R. (2017). Neuronal Decanoic Acid Oxidation Is Markedly Lower than That of Octanoic Acid: A Mechanistic Insight into the Medium-Chain Triglyceride Ketogenic Diet. Epilepsia.

[52] Lee N., Sa M., Hong Y.R., Lee C.J., Koo J. (2018). Fatty Acid Increases cAMP-Dependent Lactate and MAO-B-Dependent GABA Production in Mouse Astrocytes by Activating a Gαs Protein-Coupled Receptor. Exp. Neurobiol..

[53] Feldman J.L., Dittenhafer-Reed K.E., Denu J.M. (2012). Sirtuin Catalysis and Regulation. J. Biol. Chem..

[54] Das A.M. (2003). Regulation of the mitochondrial ATP-synthase in health and disease. Mol. Genet. Metab..

